# Nanoimaging of Focal Adhesion Dynamics in 3D

**DOI:** 10.1371/journal.pone.0099896

**Published:** 2014-06-24

**Authors:** Chi-Li Chiu, Jose S. Aguilar, Connie Y. Tsai, GuiKai Wu, Enrico Gratton, Michelle A. Digman

**Affiliations:** 1 Department of Developmental and Cell Biology, University of California Irvine, Irvine, California, United States of America; 2 Department of Biological Chemistry, University of California Irvine, Irvine, California, United States of America; 3 Department of Biomedical Engineering, Laboratory for Fluorescence Dynamics, University of California Irvine, Irvine, California, United States of America; King's College London, United Kingdom

## Abstract

Organization and dynamics of focal adhesion proteins have been well characterized in cells grown on two-dimensional (2D) cell culture surfaces. However, much less is known about the dynamic association of these proteins in the 3D microenvironment. Limited imaging technologies capable of measuring protein interactions in real time and space for cells grown in 3D is a major impediment in understanding how proteins function under different environmental cues. In this study, we applied the nano-scale precise imaging by rapid beam oscillation (nSPIRO) technique and combined the scaning-fluorescence correlation spectroscopy (sFCS) and the number and molecular brightness (N&B) methods to investigate paxillin and actin dynamics at focal adhesions in 3D. Both MDA-MB-231 cells and U2OS cells produce elongated protrusions with high intensity regions of paxillin in cell grown in 3D collagen matrices. Using sFCS we found higher percentage of slow diffusing proteins at these focal spots, suggesting assembling/disassembling processes. In addition, the N&B analysis shows paxillin aggregated predominantly at these focal contacts which are next to collagen fibers. At those sites, actin showed slower apparent diffusion rate, which indicated that actin is either polymerizing or binding to the scaffolds in these locals. Our findings demonstrate that by multiplexing these techniques we have the ability to spatially and temporally quantify focal adhesion assembly and disassembly in 3D space and allow the understanding tumor cell invasion in a more complex relevant environment.

## Introduction

The study of focal adhesions in the two-dimensional (2D) environment has led to an in depth understanding of their protein composition [1], structure [2], and their role in cell migration as well as mechanical sensing. Focal adhesions connect extracellular matrix (ECM) and F-actin cytoskeleton through transmembrane protein integrins [3]–[6]. Feedback interactions from mechanical and biochemical signals within focal adhesion and the F-actin cytoskeleton coordinate the behavior of the protrusive and contractile lamella by promoting and sustaining the proper spatial and temporal control in the cell [3].

The formation of focal adhesions on 2D surfaces begins with integrin clustering upon interaction with the ECM. Small transient integrin-associated nascent adhesions form first, followed by the formation of larger, more stable fibrillar adhesion with actin stress fibers, which facilitate cell spreading and migration [7]. The size of focal adhesion structures ranges from <0.25 µm (nascent adhesion) with fast turnover rate of >5 µm (fibrillar adhesion) with slower turnover rates [3], [8]. Whether focal adhesions form in the 3D environment is still under debate [9]–[12]. It has been postulated that focal adhesions may not form at all due to the pliability of the microenvironment [11]. In addition, when cells are in the 3D environment, there is a continuum of migration modes that are determined by both matrix substrate and intrinsic contractility of the cell [7], and focal adhesions may not be needed for migration. The discrepancy of cellular migratory behavior, when focal adhesion-related components in 2D and 3D are altered, could indicate that focal adhesions in 3D, if they exist, may carry out different roles [12]–[14].

Focal adhesions are most commonly visualized in 3D using immunofluorescence staining [9]. By this method, several groups have reported the existence of focal adhesions in metastatic human breast cancer cell line, MDA-MB-231, either cultured in Matrigel [15] or type I collagen matrix [16]. These focal adhesions are found on cell protrusions close to the tip. However, immuno-staining prevents investigations to probe protein dynamics and suffers from possible artifacts due to sample fixation. Finding focal adhesion sites in live cells embedded in 3D matrices has been challenging. Compared to 2D imaging, conventional confocal microscopes have an axial resolution that is about three times lower than lateral resolution, which makes it difficult to discern very small structures such as focal adhesions. In addition, current laser scanning confocal microscopy uses a predetermined raster scan pattern to move the laser spot for imaging one plane at a time. This is inefficient to image structures that are sparse in 3D such as a cell protrusion. Due to the longer acquisition time required for 3D imaging, protein dynamics that occur in short timescales cannot be detected. Recent literature has discussed several other issues regarding focal adhesion studies of live cells in 3D. First, the focal adhesions detected may be from the cells that experience the stiff glass surface due to the proximity to the imaging dish (‘edge effect’), which is not a true 3D environment. In this case, the cell may behave more similarly to the 2D scenario. The underlying idea is that if part of a cell can sense the glass surface, the behavior of the entire cell may be biased by the properties of the surface stiffness. Second, high cytoplasmic fluorescence background may give low signal-to-background ratio that hinders the detection of focal adhesions [10]–[12].

**Figure 1:**
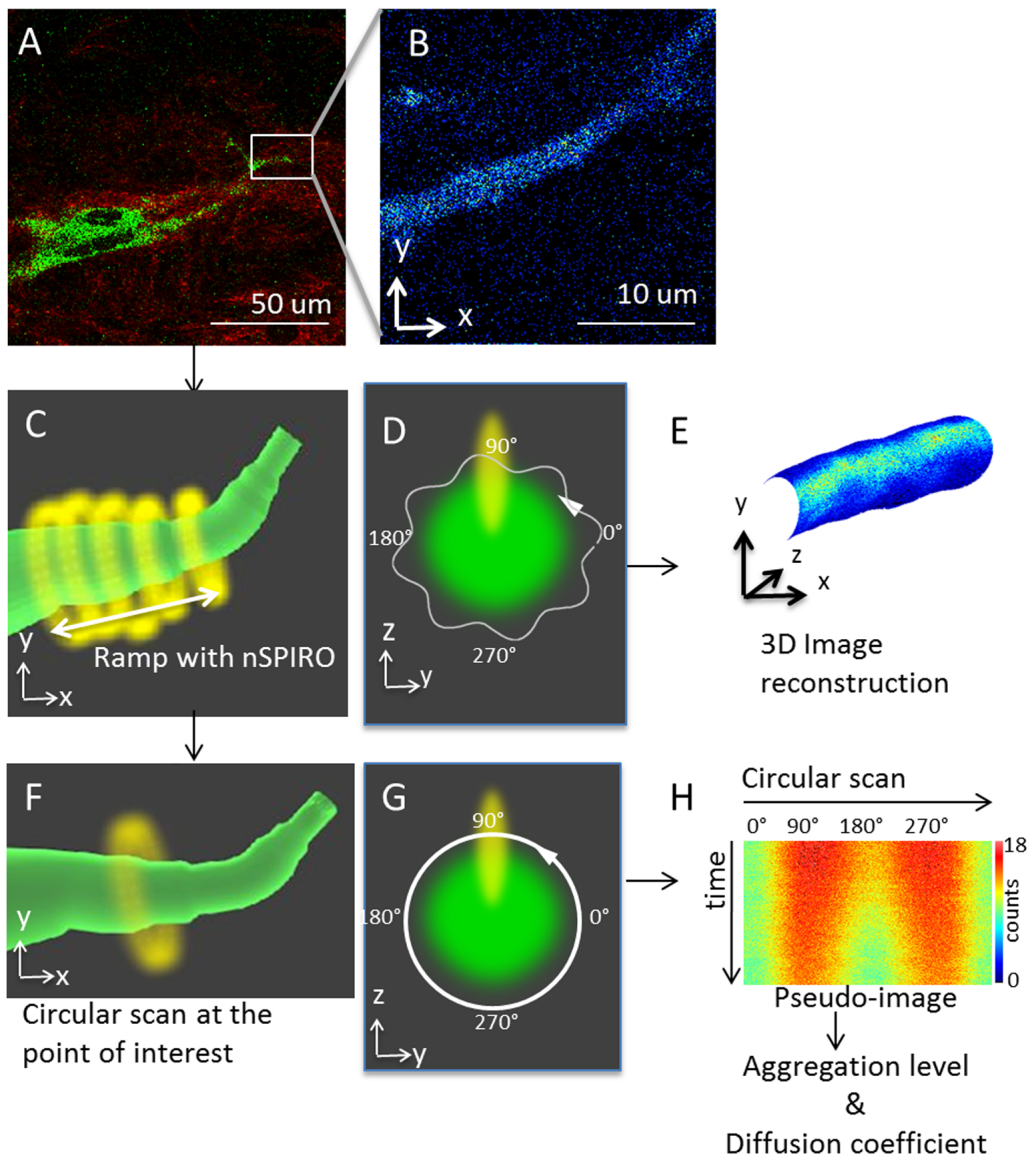
Experimental workflow. (A) First, a raster scan image of an MDA-MB-231 cell with fluorescent expression (green) cultured in type I collagen (red) was taken. In this example, MDA-MB-231 cell was labeled with paxillin-GFP, and the image was taken with 2-photon microscope to detect simultaneously fluorescence and SHG from the collagen. (B) Once the location of cell protrusion was identified, we took zoomed to identify a cell protrusion. Here only the fluorescence image is shown. (C) After identifying the cell protrusion region of interest, we switched the scanning mode from raster scan to nSPIRO. This schematic graph shows the nSPIRO circular scan (yellow) ramps along the cell protrusion (green) identified from (B). The circular scan orbit size was selected according to the cell protrusion radius. While ramping along the cell protrusion, the center position of circular scan is maintained on the cell protrusion by nSPIRO feedback method, as described in Materials and Methods section. The intensity profiles of fluorescent proteins in cell protrusion as well as SHG of nearby collagen fibers aree recorded simultaneously. (D) A schematic graph showing the cross section of the protrusion shown in (C) in green, and the trajectory of circular orbit with amplitude modulation (white). The yellow oval shape represents the PSF. The PSF is elongated along z direction, resulting in higher intensity near 90° and 270° due to larger observation volume overlap with the fluorescent structure, and lower intensity near 0° and 180°. (E) After collecting the data from (C), the 3D image reconstruction can be done. The reconstruction utilizes circular scan location and radius to create cylindrical-shaped mesh covered with a texture which represents the intensity profile. (F) Schematic graph showing that the circular scan method can also be applied at a point of interest while the nSPIRO tracking routine maintains the orbit centers on the protrusion. This technique allows the measurement of protein dynamics at a specific orbit location with high temporal resolution. (G) A schematic graph showing the cross section of the protrusion shown in (C) in green, and the trajectory of circular orbit without amplitude modulation (white). The arrow indicates the orbit scan direction, and 0° represents the orbit starting point. The yellow oval shape represents the PSF. The amplitude modulation was not used to avoid introducing artifact for image correlation analysis. (H) Circular scans data from (F) were represented as a pseudo-image in which the horizontal dimension is pixel position along a circular scan, which corresponds to the position 0° to 360° in (G), and the vertical dimension is time. In the pseudo-image, the intensities around 90° and 270° were higher than at 0° and 180° due to the PSF shape, which is different in the x and z directions, as explained in (D). The color scale shows that this effect is not very large, accounting for about 20% variations along the orbit. The data were further analyzed to retrieve protein diffusion coefficients and aggregation level, as described in Materials and Methods.

In conjunction with high-resolution 3D imaging reconstruction by nSPIRO, spatial correlation of the intensity fluctuations during the orbital scanning were used to provide the diffusion coefficients of proteins near the protrusion surface. To obtain the high temporal resolution needed to measure protein diffusion, we implemented circular scan around the cell protrusion at the region of interest, while maintaining the orbit centered at the protrusion by the nSPIRO feedback approach to avoid the fluctuation caused by cellular movement (Fig. 1F). Image correlation spectroscopy [17] and Number and Brightness (N&B) methods [18] can then be applied to the intensity trace around the protrusion to extract protein diffusion rates of focal adhesion proteins and to analyze protein aggregation level, respectively (see Materials and Methods).

In this study, we used EGFP-labeled paxillin for focal adhesion identification, a multi-domain scaffold protein that generally localizes at focal adhesions. Paxillin serves as an adaptor protein for the recruitment of numerous regulatory and structural proteins that control the dynamic changes in focal adhesion and cytoskeleton [19], [20]. The dynamics of paxillin in 2D environment has been described in detail, and has been shown that the diffusion rate as well as aggregation level are different at focal adhesion sites compared to cytosolic position [21]. For our analysis, we first showed evidence of physical attachment of the cell to collagen fibers by spatial-temporal image correlation spectroscopy (STICS) technique to map the displacement of the collagen fibers as the cells migrate in the 3D environment. The displacement of the collagen fibers as the cell moves allows us to map regions of attachment of the fibers. Second, we used the nSPIRO technique to reconstruct the surface of the cell protrusion with nanometer precision. At the cell protrusion, paxillin and α5-integrin are co-localized. Using a second detection channel to measure the second harmonic generation (SHG) signal of collagen we also found that collagen co-localize with paxillin and α5-integrin at the adhesions. Interestingly, we found that paxillin-EGFP formed aggregates on two sides of a collagen fiber, and that this structure could be stable for several minutes. Our results on the slow diffusive behavior and increase in molecular brightness of paxillin at the key site that co-localize with collagen are readily identified as a protein organization site of focal adhesions. The focal adhesions formed in 3D are smaller and have faster dynamic assembly and disassembly compared to the focal adhesions formed in 2D.

## Materials and Methods

### Cell culture preparation

The paxillin-EGFP MDA-MB-231 stable cell line (American Type Culture Collection, HTB-26) was prepared as follows. First, the paxillin gene was ligated into the pEGFP-N3 vector kindly provided by the Horwitz lab at the University of Virginia. The Paxillin-EGFP fusion was then inserted into the pQCXIP retroviral expression vector. The retrovirus was prepared using the packaging cell line GP2-293 (Clontech, Palo Alto, CA). MDA-MB-231 was infected with retrovirus for 24 hours and selected with puromycin at 1 microgram/ml for a few days to obtain the stable line.

Transient transfections of actin-EGFP, actin-mCherry were done using Lipofectamine 2000 (Invitrogen, Carlsbad, CA) according to the manufacturer's protocol. MDA-MB-231 and U2OS cells were cultured in Dulbecco's modified Eagle's medium (DMEM) with high glucose (Sigma-Aldrich, St. Louis, MO) supplemented with 10% (v/v) fetal bovine serum and 1% pen/strep at 37°C in a 5% CO_2_ incubator.

Type I collagen was purchased from BD Biosciences (Franklin Lakes, NJ), with the original concentration of 3.75 mg/ml. Collagen was diluted with 10× PBS with phenol red and water to final concentration of 1× PBS and 2 mg/ml collagen solution. NaOH was added to neutralize the collagen solution before mixing with cells. Fluorescent labeled MDA-MB-231 cells or U2OS cells in serum-free DMEM were mixed with 2 mg/ml collagen solution, with the final concentration of 5×10^4^ cells/ml. The collagen/cell mixture was polymerized at 20°C for 1 hr and then at 37°C for 20 min. Full medium was applied after polymerization and placed in the CO_2_ incubator.

Measurements were performed 2 to 4 days after cells were cultured in the collagen matrix.

### 2-Photon imaging

2-Photon raster scan images with z-stack scanning over time were obtained using LSM 710 (Carl Zeiss, Maple Grove, MN) and rendered using the ZEN software (Carl Zeiss). A Mai-Tai Ti∶Sa laser (Newport, Irvine, CA) with excitation wavelength at 900 nm was used for the raster scan images. The emission was collected by a 40×0.8 N.A. water immersion objective with working distance of 3 mm and detected using a filter (442–463 nm) for second harmonic generation for collagen and 523–646 nm filter for EGFP emission.

### nSPIRO tracking principle

The detail of nSPIRO algorithm can be found in [22]–[24]. In brief, the laser beam is moved in a circular orbit with the radius close to the fluorescent object of interest. The radius of the orbit is modulated at a frequency of 8 oscillations per period. By Fourier transform, the fluorescence signal recorded along the circular orbit gives the phase and the radial distance of the object from the center of circular scanning. The laser circular orbit center position is then updated according to the fast feedback algorithm in real time by moving the center of the orbital scanning to the recovered object position.

### nSPIRO microscopy setup

The nSPIRO tracking routine was performed on a home-built 2-photon microscope as previously described [22]. The same 40× water objective for 2-photon imaging was used. A Chameleon Ultra II laser (Coherent, CA), set to 880 nm, was used to excite EGFP. A 490 nm dichroic was used to split the emission signal into two channels. The emission for the second harmonic generation (SHG) signal from collagen was recorded simultaneously with GFP signal. The wavelength collected for SHG signal was between 387 nm to 447 nm and 515 nm and 555 nm for EGFP. All experiments involving nSPIRO were performed with the specification as described above. For co-localization experiment nSPIRO was performed on a modified inverted Olympus FV1000 microscope. Two different laser lines at 488 nm and 543 nm were used simultaneously for excitation of α5-integrin-EGFP and paxillin-mCherry, respectively. The EGFP emission was collected between 505–525 nm and the mCherry emission was collected between 560–660 nm.

Figure 1 shows the experimental design. Briefly, the cell protrusion in 3D was first located by raster scan (Fig. 1A and 1B). Then circular scan with amplitude modulation was then performed along the cell protrusion with 20 seconds to 60 seconds per ramp period (Fig. 1C). Once the ramping along the protrusion begins, the nSPIRO tracking feedback system updates the cell protrusion center position based on the fluorescence intensity acquired along the orbit. The orbit center position is adjusted every 4 orbits. At the end of the 4-orbit cycle, the orbit is moved by a small amount (generally less that 100 nm) along the axis perpendicular to orbit plane (Fig. 1C). Depending on the diameter of the cell protrusion, the orbit radius was set at different values between 1 µm to 4 µm. The fluorescence intensity was recorded either at 128 or 256 points along the orbit at a sampling rate of 16 ms/orbit. We used a radial modulation of 10% of orbit radius modulation (Fig. 1D). This modulation is sufficient to determine the position of the surface of the protrusion with nanometer accuracy. The nSPIRO data was acquired and processed by the SimFCS software (www.lfd.uci.edu, UCI, Irvine). Images from nSPIRO were rendered by SimFCS (Fig. 1E). The 3D image reconstruction utilizes orbit location and radius to create cylindrical-shaped meshes and textures which correspond to the intensity profile along each orbit.

### STICS

To analyze cell-collagen interaction at the cellular scale, we used spatial temporal image correlation spectroscopy (STICS) analysis [25], [26] on confocal raster scan images taken from LSM710 microscope. By correlating frames across time-series images, STICS gives the information of the direction and speed of flow. This method was originally developed for molecule dynamics, and has been expanded to analyze cellular sizes and movements [27]. Here we applied STICS to larger scale objects, cell protrusions and collagen fibers, to analyze their relative motions. Images were acquired with a frame size of 28.16 µm*28.16 µm (256*256 pixel) to cover the cell protrusion, and the sub-frame size used for the STICS algorithm was 7.0 µm*7.0 µm (64*64 pixel) to capture regional collagen fiber movement. Images were taken every 6 seconds for 10 minutes. The STICS algorithm is implemented in the SimFCS software.

### Diffusion and Aggregation Analysis During the Orbital Scan

To acquire dynamics at the protein level in cell protrusions, we performed circular scans around the cell protrusion while maintaining the center of mass by the nSPIRO feedback algorithm (Fig. 1F). We did not apply radial modulation (Fig. 1G) during these measurements as the purpose here is to use image correlation to measure dynamics, and radial modulation may introduce artifact. Circular scans were done at 16 ms per period, with 128 pixels acquired for each period. The radius of the circular scan was chosen to be close to the dimension of cell protrusion, between 1 µm to 4 µm. To measure protein diffusion rates, we applied image correlation spectroscopy along the scan to analyze protein dynamics [17], [21], [28]. As previously described by Digman et al. [28], the data acquired by the circular scan method are presented as an image carpet (Fig. 1H), in which each column (i axis) is the intensity along the circular orbit, and each row (j axis) represents an orbit taken at different time points. The spatial correlation function defined below was then applied to the image intensity (*I*):
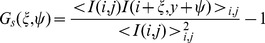
(1)where ξ and ψ are the spatial increments in the i and j directions, respectively, and the angle bracket indicates average over all the spatial locations in both i and j directions.

The spatial correlation function for circular scan is the product of two terms: a correlation term to the scanning (S) parameter and a correlation term due to diffusion (G) of the molecule:
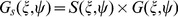
(2)For circular scan, S and G are given by:
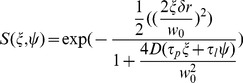
(3)

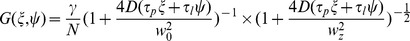
(4)In which δr is the distance between adjacent points along the orbit, and τ_p_ and τ_l_ are the pixel dwell time and time between orbits, respectively. *w*_0_ and *w*_z_ are geometrical factors that describe the laser beam profile. N is the number of particles in the focal volume, and γ depends on the shape of the illumination volume. Eq. 3 and Eq. 4 are used to fit the image correlation function from circular scanning data to extract the diffusion coefficient D. Fitting is done by the SimFCS software. A moving average of 50×256 lines (210 seconds) was used to filter out very slow motion as previously described [21], [28].

The number and brightness (N&B) method [18], [29] was applied to the same dataset to identify protein aggregates. The number and brightness of particles at each pixel of the circular scan can be extracted by using the average and the variance of the intensity distribution. For K time points with corresponding intensity k_i_, the average <k> and the variance σ^2^ of the intensity distribution are given by
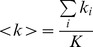
(5)

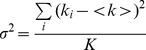
(6)According to Digman et al [29], the variance of the intensity measurement is due to the combination of the variance caused by particle fluctuation (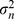) and the variance caused by the shot noise of the detector (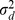). These two variables as well as the average intensity <k> are dependent on the molecular brightness, *ε*, which reflects the aggregation state of fluorescent proteins, and the average number of molecules in the illumination volume, n.
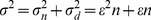
(7)

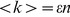
(8)The apparent brightness (B) is defined as the ratio of the variance to the average intensity at each pixel. By substituting the variance and the average to *ε* and n using Eq. 7 and Eq. 8, the value B can be shown to be related to the brightness of the particles, which could be use to determine the aggregate size. B is independent of the number of particles [29].
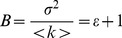
(9)We report the B value as the indication of protein the aggregation level. The N&B algorithm is implemented in SimFCS.

### Statistical analysis

For the comparison of molecular brightness at focal adhesion and non-focal adhesion sites we performed two-tail t test to calculate the P value to determine if the difference of these two groups is significant (P value<0.01).

We performed binomial test to calculate the P value to compare slow diffusive paxillin populations detected at focal versus non-focal adhesion sites.

## Results

### Morphological differences of MDA-MB-231 cells in the 2D and 3D environment and interactions with collagen

**Figure 2:**
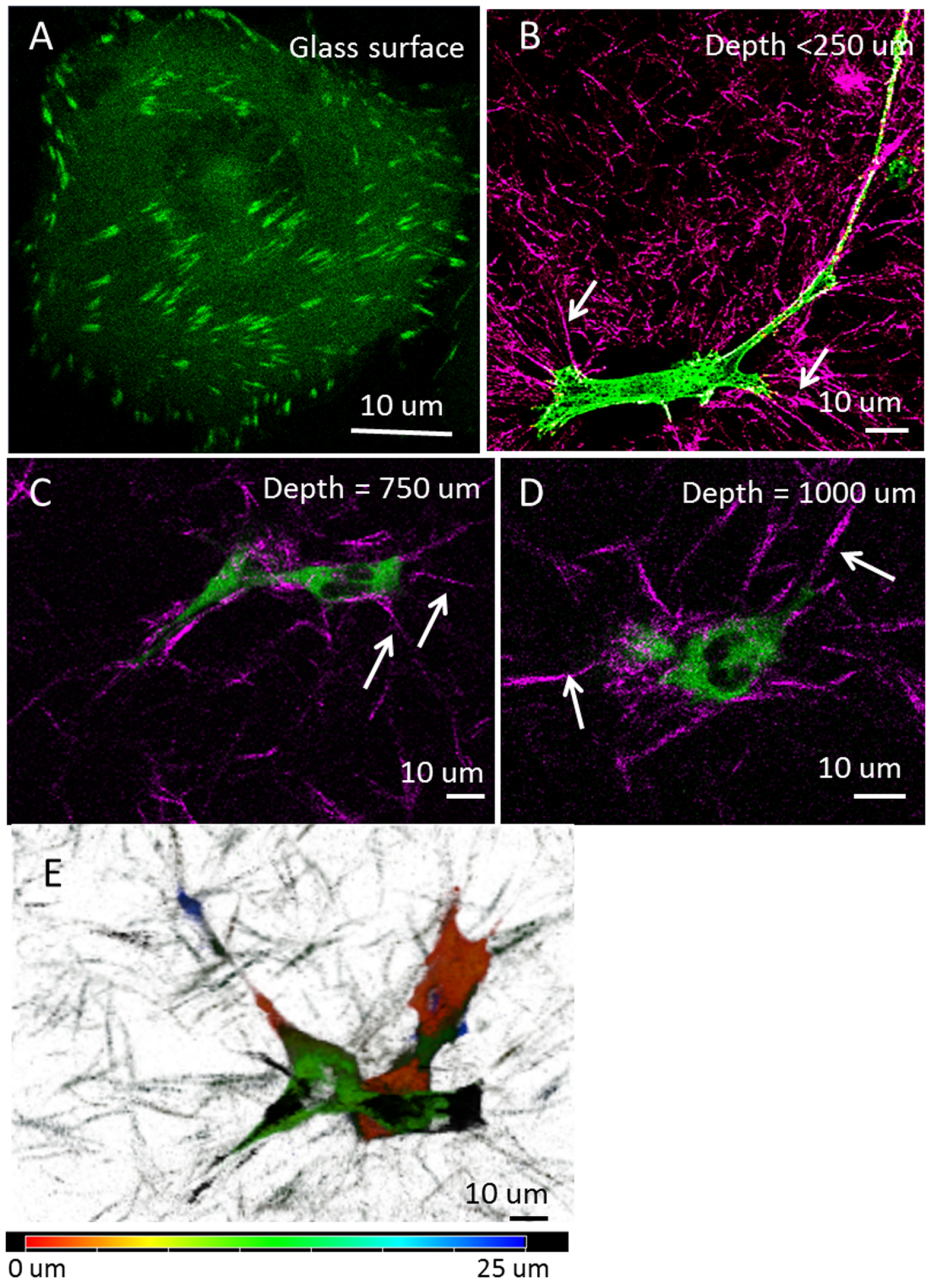
MDA-MB-231 cells in 3D type I collagen matrix. (A–D) 2-photon excitation raster scan images of MDA-MB-231 cells labeled with paxillin-EGFP (green) in type I collagen matrix (purple) at different depth from the glass surface, as indicated in each panel. Cells on the glass surface show focal adhesions at the cell borders (A), but there are no distinctive focal adhesions when cells are in collagen matrix (B–D, depth is the distance to the glass surface). However, collagen fibers were remodeled near the cells, showing more straight fibers toward the cell body as indicated by the white arrows, and collagen fibers away from cells were more randomly distributed, as shown in panel B. (E) Depth color-coding showing that MDA-MB-231 cells embedded in collagen matrix (750 µm above glass) have protrusions across tens of micrometers in depth, but the major cell bodies were about the same height.

MDA-MB-231 cells expressing paxillin-EGFP were cultured on either 2D culture dishes or embedded in type I collagen matrices for 2 to 4 days before imaging. In contrast to the flat spreading morphology on 2D culture dish surfaces with distinct focal adhesions near the cell border (Fig. 2A), MDA-MB-231 cells either stay rounded or form long protrusions when cultured in collagen matrices, as previously reported [16], [30]. Using 2-photon laser scanning microscopy, no distinct focal adhesion was detected in the collagen matrix samples (Fig. 2B–D). However, we observed cell protrusions actively forming and retracting. In addition, collagen deformation was also found near cell protrusions, where collagen fibers are stretched toward the cell (Fig. 2B–D). This phenomenon is ubiquitous in collagen matrices close to the glass surface up to about 1 mm above the glass. Interestingly, cell protrusions in 3D were not randomly growing in all directions. Although the entire cell could span up to about 100 µm, the body of the cell was mainly aligned parallel to the glass surface on the xy plane (Fig. 2E).

**Figure 3:**
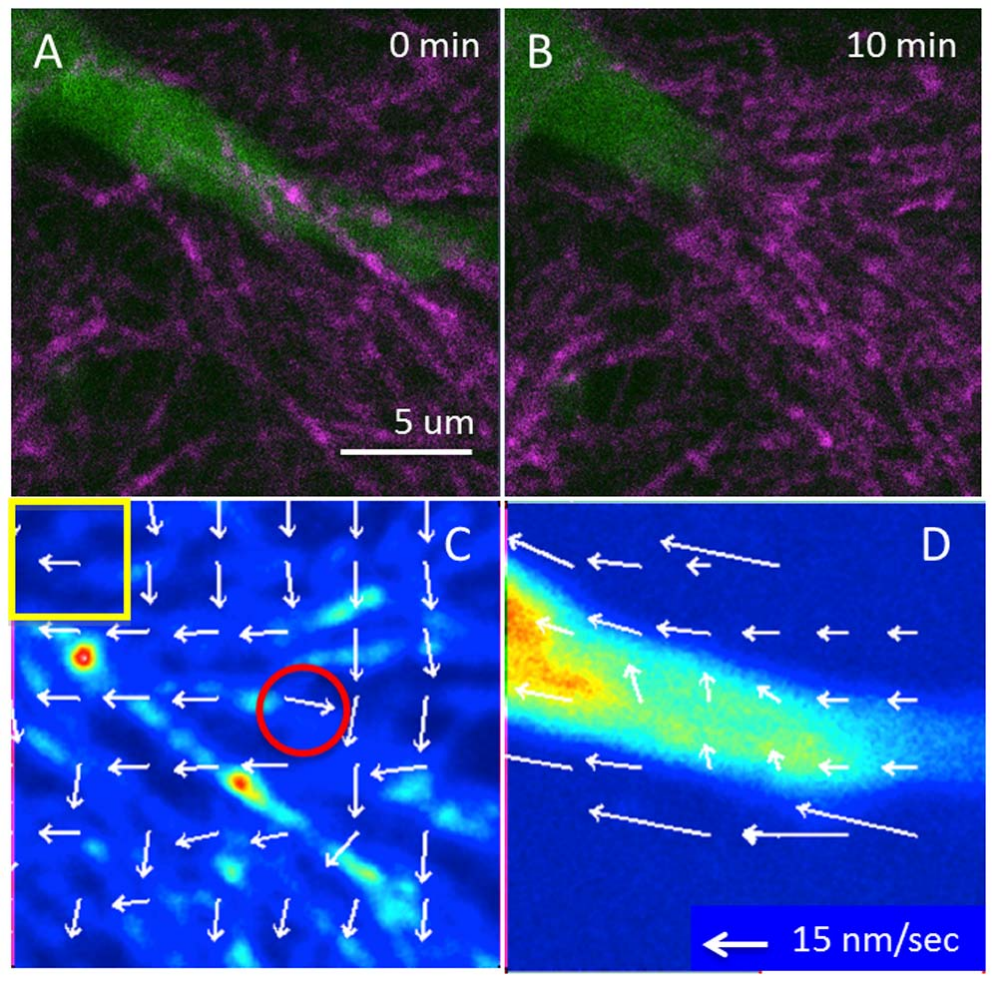
STICS analysis showing collagen fiber displacement. (A and B) STICS analysis was applied to a 10 minutes time-lapse movie of collagen SHG signal (purple) and MDA-MB-231 cells with actin-EGFP expression (green) taken by the two-photon microscope to collect the SHG of collagen. (A) Shows the beginning and (B) shows the end of the time-lapse. (C and D) STICS was applied to 64×64 pixel regions on the entire 256×256 pixels image for both channels. (C) Shows STICS analysis on collagen. The yellow box shows the size of the sliding region used for analysis. Arrows indicate the speed and the direction of the average motion at each 32×32 pixel region. The red circle shows a region of collagen fiber that moved toward the opposite side of cell retraction, indicating possible detach and relaxation of collagen fiber from the cell. (D) Shows the SITCS analysis on the fluorescence signal form the cell protrusion, which has more uniform movement.

To establish whether the interaction of cell protrusions and collagen fibers was passive or involved focal adhesions, the STICS analysis was applied to time-lapse images of an MDA-MB-231 cell embedded in type I collagen (see Materials and Methods, Fig. 3). SHG signal from collagen and actin-EGFP emission were simultaneously collected. Within the 10-minute data acquisition, the cell protrusion showed significant retraction towards the upper-left corner of the image (Fig. 3A and 3B). The STICS analysis further revealed the local deformation of collagen fibers and cell protrusions. Figure 3C and figure 3D show STICS velocity maps of collagen and cell protrusion, respectively. Collagen fibers near the cell protrusion were moving in the same direction of cell retraction, which may due to passive squeezing. However, while some collagen fibers were moving along the direction of motion of the protrusion there was one point in time and space where the collagen detached from the cell and snapped-back in the opposite direction (Fig. 3C, red circle), suggesting the existence of adhesions.

### Visualizing focal adhesion on cell protrusion

To visualize focal adhesions in 3D, we applied the nSPIRO technique [22] to achieve higher spatial-temporal resolution at cell protrusionscompared to 3D confocal imagesand to demonstrate the existence of focal adhesions in both MDA-MB-231 cells and U2OS cells. The measurements were done on cells embedded in collagen matrices and away from the imaging dish glass edge, avoiding previously discussed ‘edge effect’ due to proximity of the glass [11].

**Figure 4:**
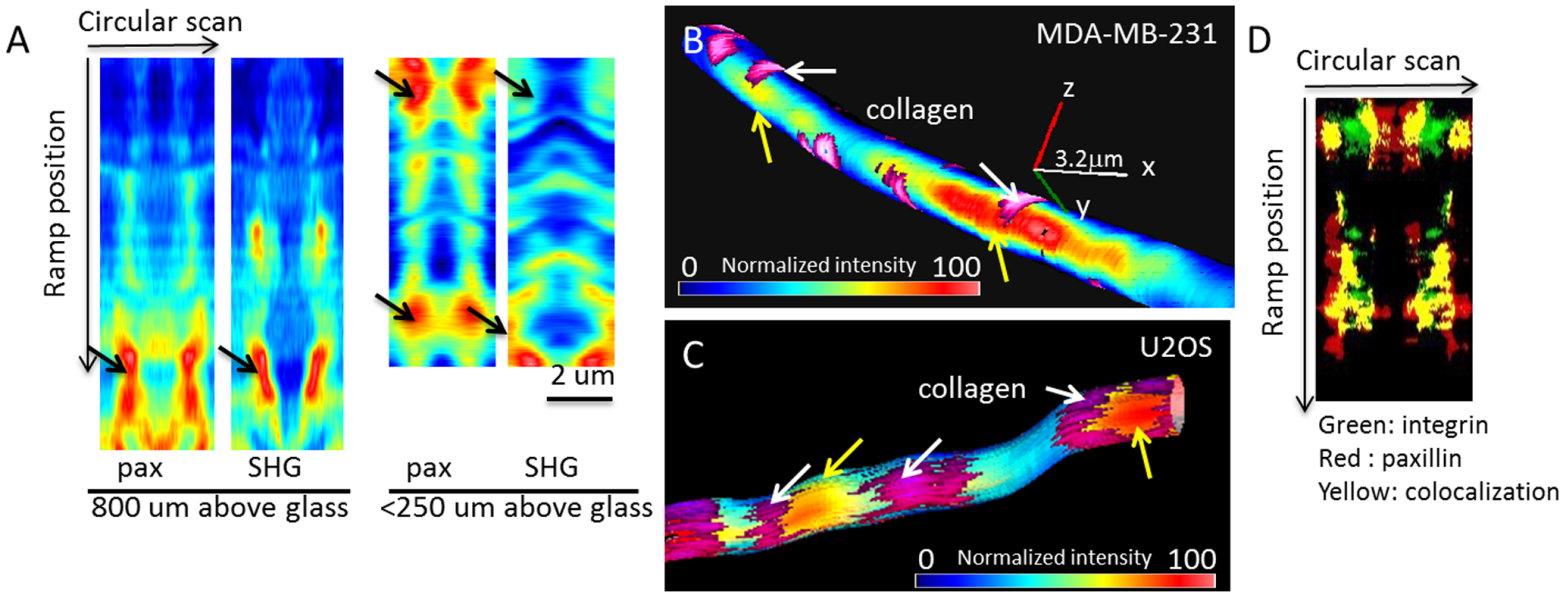
Paxillin localization detected by nSPIRO imaging. (A) Paxillin-EGFP (pax) and collagen SHG intensity measured by nSPIRO along a cell protrusion as described in Figure 1C. Data were represented as pseudo-images, where the horizontal axis represents intensity along each circular scan and the vertical axis represents the ramp position along the cell protrusion. The non-uniform distribution of paxillin-EGFP was found at both cell protrusion embedded in collagen matrix (800 µm above glass) and closer to glass surface (<250 µm above glass). Furthermore, the position of high paxillin-EGFP intensity is adjacent to collagen fibers, as indicated by black arrows. (B) Nano-imaging of paxillin-EGFP expression on MDA-MB-231 cell protrusion is reconstructed as described in the introduction and Figure S1. Paxillin-EGFP intensity was color-coded and overlaid with collagen fibers (purple, indicated by white arrows) near the cell protrusion. Paxillin-EGFP shows high intensity spots (yellow arrows) on both sides of the collagen fiber, indicating the cell protrusion may ‘grab’ the collagen fiber. The axis represents 3.2 µm on x, y and z directions. (C) Nano-imaging of paxillin-EGFP expression on U2OS cell protrusion. Similar paxillin distribution as seen from MDA-MB-231 cells can be also seen in U2OS cells. (D) Integrin-EGFP (green) and paxillin-mCherry (red) showed high colocalization (yellow) at MDA-MB-231 cell protrusion, supporting the hypothesis that the paxillin high intensity sites are possible locations of focal adhesions, and the focal adhesions in 3D may be integrin-dependent.

By the nSPIRO method, we were able to visualize non-uniform paxillin-EGFP distribution in cells close to the glass surface (<100 µm distance from the surface). These non-uniform distributions where are detected in cells entirely embedded in the matrix (>900 µm above the glass surface) (Fig. 4A), in contrast to the report made by Fraley et al. [11]. Furthermore, the location of high intensity paxillin-EGFP correlates with collagen SHG intensity. As illustrated in figure 1D, the nSPIRO circular acquisition on the cell protrusion was done on the x-z or y-z plane. The intensity is modulated along the orbit due to the point spread function (PSF) profile that has larger extension along the z direction (Fig. 1D, shown in yellow). The intensity is higher when the PSF is at 90° and 270° of the orbit since the PSF penetrates more inside the protrusion, while the intensity is lower at 0° and 180° since the orbit is moving outside the protrusion. Thus, the difference in extension of the PSF along z-axis with respect to the x or y direction gives a characteristic banded pattern on the pseudo-image carpet, while the intensity along the ramping direction is not modulated. Below we describe the intensity variation along the ramping direction for the identification of focal adhesion positions.

Figure 4B and 4C shows the 3D rendering of paxillin-EGFP expression overlaid with collagen fibers in an MDA-MB-231 and an U2OS cell protrusion, respectively. Unlike images obtained with the raster scan method that showed no distinct features of paxillin-EGFP distribution in 3D, nSPIRO reveals that paxillin-EGFP was non-uniformly distributed along cell protrusions with high intensity spots, indicating the possible location of focal adhesions in 3D. These high intensity spots extend few hundred nanometers along the cell protrusion. Further analysis of paxillin-EGFP and collagen intensity showed that the local maxima of paxillin-EGFP intensity (possibly focal adhesions) are usually co-localized with collagen fibers. We found that collagen fibers at these locations are perpendicular to the direction of the protrusion. We observed that paxillin sometimes exhibits relatively symmetrical high concentration at both sides of the collagen fiber (along the protrusion axis), showing that focal contacts surround or ‘grab’ the perpendicular collagen fiber (Fig. 4B). This organization of paxillin is distinct from the shape of focal adhesions found in cells cultured on 2D surfaces. The high intensity collagen signal does not always correlate with high paxillin-EGFP signal, indicating that a nearby collagen fiber does not guarantee the formation of focal adhesions. Similar features were also seen in U2OS cell protrusions (Fig. 4C). We further performed α5-integrin-EGFP and paxillin-mCherry co-transfection experiment to show that at these high intensity spots both proteins are present. We used one photon nSPIRO as described in *nSPIRO microscopy setup* under *Materials and Methods*, and showed co-localization of α5-integrin and paxillin in MDA-MB-231 cell protrusions at the high intensity spots shown in yellow pixels (Fig. 4D).

**Figure 5:**
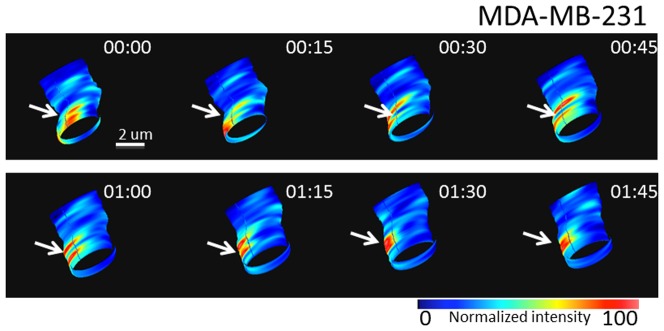
Time-lapse images of paxillin-EGFP at cell protrusions. Time-lapse 3D images from nSPIRO acquisition of an MDA-MB-231 cell protrusion with 15 seconds interval showing the dynamics of paxillin-EGFP. Focal adhesions (high paxillin-EGFP intensity region) were constantly changing the position, as indicated by white arrows.

The speed of the nSPIRO data acquisition allows us to generate 3D movies of the cell protrusion with ‘frame’ rate in the seconds scale. Figure 5 shows paxillin-EGFP dynamics on an MDA-MB-231 cell protrusion and demonstrates that the paxillin-EGFP high intensity spots can last for minutes, although the position may change. We speculate that the position change might be related to the collagen fiber movement.

### Protein diffusion and binding

**Figure 6:**
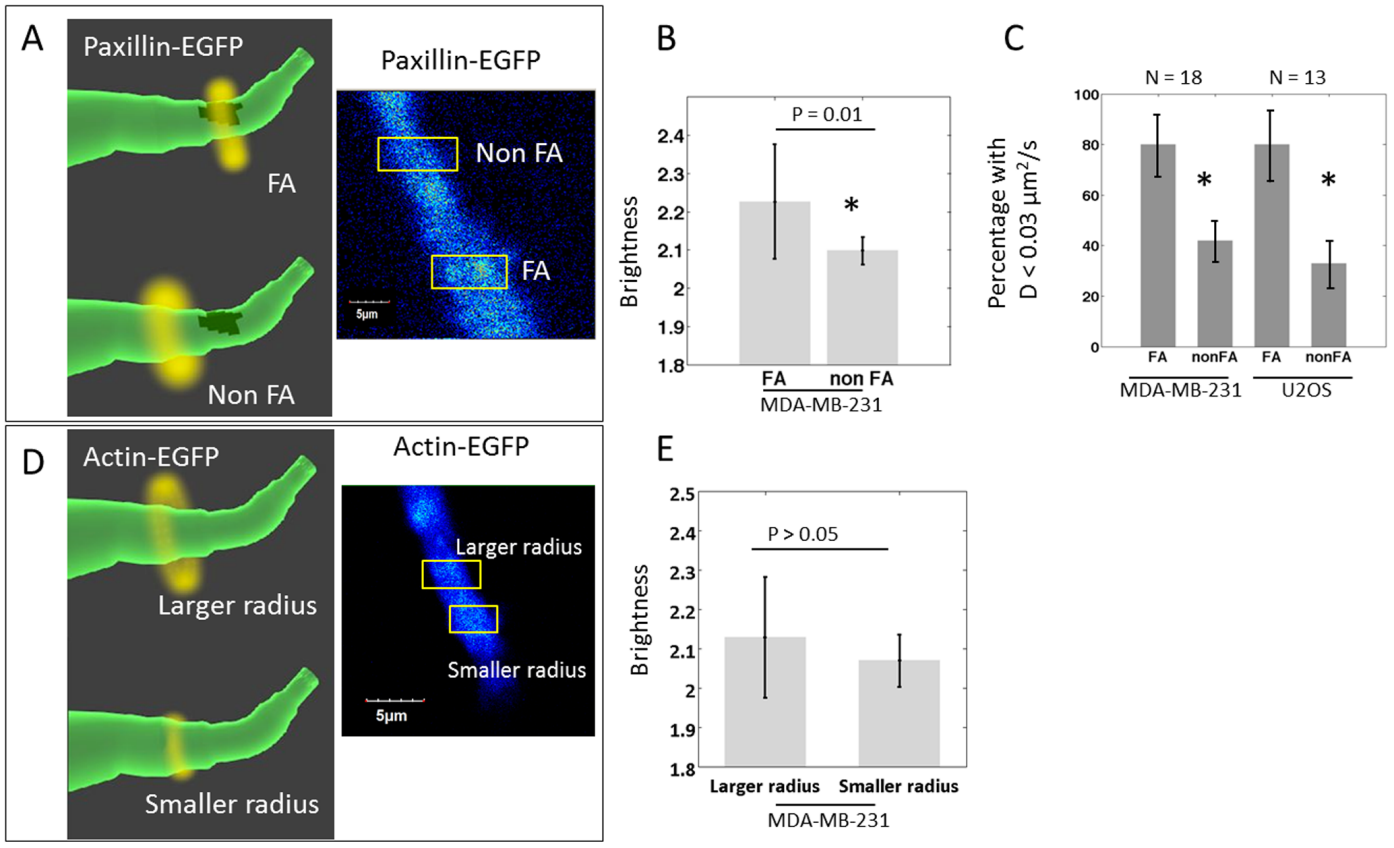
Aggregation and diffusion coefficient differences of paxillin-EGFP and actin-EGFP at FA and non FA. (A) Schematic diagram and actual image showing the principle of the measurement of paxillin-EGFP dynamics at high intensity region (focal adhesion, FA) and low intensity region (non-focal adhesion, non FA). This measurement provides the aggregation level and diffusion coefficients of the protein. The method was described in Figure 1F and 1H as well as in the Materials and Methods section. (B) Using N&B analysis, paxillin-EGFP brightness measured at FA and non FA locations on MDA-MB-231 cell protrusion were calculated. The brightness, which reflects the aggregation level, of paxillin-EGFP from FA is higher than the brightness from non FA, indicating the presence of paxillin aggregates at focal adhesion in 3D. (C) Although at both FA and non FA locations the free diffusive paxillin can be found, the very slow diffusion population (D<0.03 µm^2^/s) was identified more frequently in FA location than non FA location for both MDA-MB-231 and U2OS cell lines. (D) Schematic diagram and actual image showing the measurement of actin-EGFP dynamics at cell protrusions with larger radius (∼2 µm, close to the dimension of cell protrusion) and with smaller radius (0.7 µm). (E) MDA-MB-231 cell actin-EGFP brightness value (reflecting the aggregation level) measured with larger and smaller radius do not show significant difference. However, as mentioned in the result, the actin diffusion rate measured was significantly slower than paxillin, indicating the existence of actin polymers or actin binding/unbinding events.

To further confirm that stable paxillin-EGFP high intensity positions are focal adhesion sites, we applied circular scan on cell protrusion at focal adhesion sites and non-focal adhesion sites. Fluctuation correlation analysis was then applied to characterize protein dynamics as described in the Materials and Methods sections (Fig. 6A). Briefly, the protein diffusion rate was determined by applying image correlation analysis to circular scanning intensity carpet pseudo-images. The spatial correlations functions was fit with a diffusion model as described in the method section. For the same dataset, we also estimated the relative protein aggregation level by the N&B method.

When comparing the N&B results obtained from paxillin-EGFP low intensity regions versus high intensity regions, on the same MDA-MB-231 cell protrusions, the stable high intensity regions showed significantly larger aggregates as indicated by higher brightness (Fig. 6B). This observation further confirms the existence of focal adhesions in 3D protrusion.

To estimate the diffusion coefficient, the fit was done using two diffusion components model. For MDA-MB-231 cells, at both paxillin-EGFP high intensity (focal adhesion, FA) and low intensity (non focal adhesion, non FA) regions, three major diffusion coefficient populations were observed: fast diffusion population (D = 22±1.1 µm^2^/s for FA and D = 23±2.8 µm^2^/s for non FA), slow diffusion population (D = 2.0±0.7 µm^2^/s for FA and D = 2.1±0.8 µm^2^/s for non FA), and very slow diffusion population (D = 0.024±0.010 µm^2^/s for FA and D = 0.024±0.008 µm^2^/s for non FA). The last component is much more prominent in FA regions compared to non FA regions (Fig. 6C, P value<0.01 by binomial test). At FAs, 80% of the measurements showed diffusion coefficient smaller than 0.03 µm^2^/s, while at non FAs only less than half of the instances showed the existence of very slow diffusion component. A similar trend was also observed with the U2OS cells (P value<0.01 by binomial test). For U2OS cells, the fast diffusion population has diffusion coefficient of 25±2.3 µm^2^/s and the slow diffusion population (D<0.03 µm^2^/s) is frequently observed at focal adhesion sites (Fig. 6C). The result is similar to paxillin dynamics in 2D cells analyzed by the RICS method [21], in which the reported free diffusive coefficient is 19.6 µm^2^/s and the slower diffusion coefficient is 1.43 µm^2^/s. In the 3D case, while the slow population (D∼2 µm^2^/s) may represent paxillin binding/unbinding at the adhesions, the last category (D∼0.02 µm^2^/s) could be the diffusion near stable focal adhesions.

We focused on whether actin has different dynamics inside the protrusion and closer to the surface of the cell protrusion. This was done by changing the radius of the orbit as to penetrate more or less the protrusion. At either location, the actin diffusion coefficient measured by the image correlation method is significantly slower (D<0.02 µm^2^/s), and is also slower than the actin diffusion rate in 2D reported in the literature (∼3.1 to 5.8 µm^2^/s) [31]. The slow diffusion may due to relatively stable actin structures in cell protrusions, which supports the hypothesis that focal adhesions exist in 3D cell protrusions. To confirm the difference of paxillin and actin diffusion coefficients, MDA-MB 231 cells were co-transfected with actin-EGFP and paxillin-mCherry. The result from the co-transfections was consistent with the results obtained from separate transfections. On the other hand, there is no significant aggregation difference for actin between regions close to the surface or in the interior of the cell protrusions (Fig. 6E) probably due to the abundance of endogenous G-actin.

## Discussion

In this work we used an array of imaging techniques to visualize and to measure the dynamics of focal adhesions in 3D. Our ability to detect focal adhesions in 3D was made possible due to the long working distance objective (3 mm) with high numerical aperture and by the nSPIRO method. Here we show that the major advantages of the nSPIRO technique over the conventional raster scan for 3D imaging are the increased resolution, more efficient acquisition, and the ability to detect fluorescence fluctuations near the object surface. The nSPIRO substantially reduces the high background fluorescence in thin cell protrusions that has been problematic for conventional confocal microscopy [10], [11]. Since the nSPIRO method enabled us to visualize focal adhesions in 3D without reducing the EGFP construct expression level, the overall signal to background ratio is large enough to visualize focal adhesions above 250 µm from the glass surface, which was difficult with conventional confocal microscope systems [11]. We also showed that nSPIRO works with one or multiphoton excitation which gave us the opportunity to observe SHG from the collagen fibers, although 2-photon excitation without the nSPIRO cannot resolve focal adhesions in 3D (Fig. 2B–D).

We used the 3D rat tail type I collagen ECM model to produce a discontinuous fibrillar structure, providing both an adhesive substrate as well as a steric barrier for cells, similar to collagen-rich interstitial tissues in vivo [32]. We found that focal adhesions in 3D occur at contacts of cell protrusion with collagen fibers, which is consistent with a previous report [10]. We demonstrated the existence of cell-collagen attachment by STICS analysis. While current traction force microscopy infers the 3D matrix deformation by measuring the displacement of beads in the matrix [33], STICS analysis may be a complementary method that gives a direct measurement of cell and collagen fiber movement. We further showed the co-localization of paxillin and collagen fibers by the nSPIRO method. Compared to the focal adhesion sizes measured in 2D cell cultures, which can be as large as 5 µm [3], [8], focal adhesions formed in the 3D environment are smaller, mostly within 2 µm (Fig. 4). This can possibly be due to a smaller surface for attachment and less tension in the collagen matrix with respect to flat surfaces [3]. Interestingly, from the distribution pattern of the focal adhesions, cell protrusions seem to run perpendicular rather parallel to collagen fibers. The co-localization of paxillin and α5-integrin as shown in figure 4D, suggests that focal adhesions in 3D may also be integrin-based. This finding establishes the 2D focal adhesion analogous in 3D.

In 3D, paxillin has been shown to regulate focal adhesion assembly/disassembly and affect cell plasticity to switch from amoeboid to mesenchymal mode of migration [34]. However, the dynamics of paxillin in the 3D environment has not been previously reported. We found three apparent diffusion coefficients of paxillin-EGFP in cell protrusion, representing free paxillin and possible paxillin binding/unbinding dynamics on focal adhesion sites. The paxillin diffusion coefficients in 3D are similar to 2D measurement [21]. However, we also observed that the slower paxillin dynamics may occur everywhere within cell protrusions, although the very slow diffusive component was found significantly more often in the FA regions. It is possible that binding/unbinding events near the cell membrane and steric hindrance play a more prominent role in determining paxillin dynamics in cell protrusions in 3D. We further demonstrated that paxillin aggregates at focal adhesions in 3D using the N&B method. The relatively small aggregation level at focal adhesions in 3D with respect to aggregates found in 2D [18] may be related to their smaller size.

In 2D, protrusions are stabilized by focal adhesions that link the actin cytoskeleton structure to the underlying ECM proteins, and acto-myosin contraction generates traction forces on the substrate that facilitates cell migration [7]. It is less known how the actin filaments are organized in the 3D environment and their interaction with focal adhesions. The actin-EGFP apparent diffusion coefficient measured in cell protrusions was significantly slower than the diffusion of paxillin-EGFP, indicating the existence of actin polymers. Other cytoskeletal components may also be involved in focal adhesion dynamics and cell motility. At least in fibroblasts, microtubules have been shown to provide mechanical structure for matrix contraction under low cell-matrix tension, whereas cells in a high cell-matrix tension state utilize conventional acto-myosin activity for matrix remodeling [35]. Further efforts to show the dynamics of actin polymerization along the protrusions are under way in our lab.

We note that under a relatively homogeneous environment without chemical stimulants while cell protrusions are actively protruding and retracting and remodeling collagen nearby, the position of cell nucleus was barely changed. From this study, it is difficult to draw conclusions whether focal adhesions are necessary for cell migration in 3D. A closer look into forces generated by the cells through focal adhesions, possibly through the STICS method, may be needed to clarify the role of focal adhesions in 3D.

Here we showed focal adhesion protein dynamics of two human cancer cell lines, MDA-MB-231 cells and U2OS cells, embedded in type I collagen matrices. These dynamics may be specific to a certain condition as it is known that both extracellular matrix and cell types affect cell migration mode [36] [37] [38]. Even within the same collagen matrix, we observed both mesenchymal-like cells with long protrusion and amoeboid-like cells with rounded or irregular shape. In type I collagen matrices, long cell protrusions seem to be the prominent feature of MDA-MB 231 cells and U2OS cells. On the other hand, when invading 3D Matrigel, Poincloux et al. observed that the same cell line has a characteristic rounded morphology with F-actin and myosin-IIa accumulating at the cell rear in a uropod-like structure [39]. MDA-MB-231 cells display neither lamellipodial nor bleb extensions at the leading edge and do not require Arp2/3 complex activity for 3D invasion in Matrigel [39]. These observations show the complexity of 3D migration, and more efforts in probing the dynamics of cell-matrix interaction may be needed for the understanding of cell migration in 3D.

In conclusion, we showed the application of the nSPIRO method combined with FCS techniques, and we demonstrated, at the molecular level, the existence of focal adhesions in 3D in which at least 3 crucial components (paxillin, a5-integrin and collagen) are co-localized and show dynamics compatible with binding/unbinding equilibria. We have demonstrated the existence of focal adhesions in cells grown in 3D collagen matrices close to the glass and in regions far away from the glass surface. We showed evidence of collagen-cell protrusion adhesions by the STICS analysis as in figure 3, and further visualized the small and sparse focal adhesions in 3D through the nSPIRO 3D imaging method (Fig. 4). We have found that paxillin diffuses with similar rates in 2D and 3D focal adhesion regions, but with higher percentage of slow diffusion population at focal adhesion sites in 3D. However, the paxillin aggregates detected are significantly smaller in the 3D environment with respect to 2D.

## Supporting Information

Figure S1 **Comparison of raster scan and nSPIRO.** (A) The raster scan pattern for 3D z-stack imaging. At each xy plane (blue), the laser beam scans line by line (black arrows) to acquire the intensity. Then the laser beam moves to a different z position and repeats the same raster scan pattern to acquire the image for another xy plane. (B) After the image is acquired as described from (A), the 3D image is reconstructed by stacking images of each xy plane at different z positions together. The grid represents pixels. As shown in this example, for a 3D sparse structure, there could be a large amount of pixels containing no information of the object of interest (green). (C) The orbital scanning pattern for nSPIRO 3D imaging. Instead of scanning line by line as raster scan, nSPIRO uses orbital laser pattern for image acquisition. With the feedback algorithm, the center of orbit scanning is adjusted to maintain the orbit on the object of interest. (D) nSPIRO orbital scan contains the information of structure size, which is used to create the mesh (black grids), and the intensity acquired along the orbit is painted on the mesh for image reconstruction. With this method, all the pixels acquired are from the object of interest, which increases the data acquisition efficiency compared to raster scan.
(TIF)

## References

[1] Kuo JC, Han X, Hsiao CT, Yates JR Iii, Waterman CM (2011) Analysis of the myosin-II-responsive focal adhesion proteome reveals a role for beta-Pix in negative regulation of focal adhesion maturation. Nat Cell Biol 13: 383–393. 21423176 10.1038/ncb2216 PMC3279191

[2] Kanchanawong P, Shtengel G, Pasapera AM, Ramko EB, Davidson MW, et al (2010) Nanoscale architecture of integrin-based cell adhesions. Nature 468: 580–584. 21107430 10.1038/nature09621 PMC3046339

[3] Gardel ML, Schneider IC, Aratyn-Schaus Y, Waterman CM (2010) Mechanical integration of actin and adhesion dynamics in cell migration. Annu Rev Cell Dev Biol 26: 315–333. 19575647 10.1146/annurev.cellbio.011209.122036 PMC4437624

[4] Geiger B, Spatz JP, Bershadsky AD (2009) Environmental sensing through focal adhesions. Nature Reviews Molecular Cell Biology 10: 21–33. 19197329 10.1038/nrm2593

[5] Geiger B, Bershadsky A (2001) Assembly and mechanosensory function of focal contacts. Current Opinion in Cell Biology 13: 584–592. 11544027 10.1016/s0955-0674(00)00255-6

[6] Hayakawa K, Tatsumi H, Sokabe M (2008) Actin stress fibers transmit and focus force to activate mechanosensitive channels. Journal of Cell Science 121: 496–503. 18230647 10.1242/jcs.022053

[7] Parsons JT, Horwitz AR, Schwartz MA (2010) Cell adhesion: integrating cytoskeletal dynamics and cellular tension. Nat Rev Mol Cell Biol 11: 633–643. 20729930 10.1038/nrm2957 PMC2992881

[8] Webb DJ, Parsons JT, Horwitz AF (2002) Adhesion assembly, disassembly and turnover in migrating cells - over and over and over again. Nature Cell Biology 4: E97–E100. 11944043 10.1038/ncb0402-e97

[9] Harunaga JS, Yamada KM (2011) Cell-matrix adhesions in 3D. Matrix Biol 10.1016/j.matbio.2011.06.001 PMC3191245 21723391

[10] Kubow KE, Horwitz AR (2011) Reducing background fluorescence reveals adhesions in 3D matrices. Nat Cell Biol 13: 3–5 author reply 5–7. 21173800 10.1038/ncb0111-3 PMC3083631

[11] Fraley SI, Feng YF, Wirtz D, Longmore GD (2011) Reply: reducing background fluorescence reveals adhesions in 3D matrices. Nature Cell Biology 13: 5–U254. 10.1038/ncb0111-3 PMC3083631 21173800

[12] Fraley SI, Feng Y, Krishnamurthy R, Kim DH, Celedon A, et al (2010) A distinctive role for focal adhesion proteins in three-dimensional cell motility. Nat Cell Biol 12: 598–604. 20473295 10.1038/ncb2062 PMC3116660

[13] Meyer AS, Hughes-Alford SK, Kay JE, Castillo A, Wells A, et al (2012) 2D protrusion but not motility predicts growth factor-induced cancer cell migration in 3D collagen. Journal of Cell Biology 197: 721–729. 22665521 10.1083/jcb.201201003 PMC3373410

[14] Kraning-Rush CM, Carey SP, Califano JP, Smith BN, Reinhart-King CA (2011) The role of the cytoskeleton in cellular force generation in 2D and 3D environments. Phys Biol 8: 015009. 21301071 10.1088/1478-3975/8/1/015009 PMC3138199

[15] Yu XZ, Machesky LM (2012) Cells Assemble Invadopodia-Like Structures and Invade into Matrigel in a Matrix Metalloprotease Dependent Manner in the Circular Invasion Assay. PLoS One 7. 10.1371/journal.pone.0030605 PMC3275555 22347388

[16] Deakin NO, Turner CE (2011) Distinct roles for paxillin and Hic-5 in regulating breast cancer cell morphology, invasion, and metastasis. Molecular Biology of the Cell 22: 327–341. 21148292 10.1091/mbc.e10-09-0790 PMC3031464

[17] Rossow MJ, Sasaki JM, Digman MA, Gratton E (2010) Raster image correlation spectroscopy in live cells. Nat Protoc 5: 1761–1774. 21030952 10.1038/nprot.2010.122 PMC3089972

[18] Digman MA, Wiseman PW, Choi C, Horwitz AR, Gratton E (2009) Stoichiometry of molecular complexes at adhesions in living cells. Proceedings of the National Academy of Sciences of the United States of America 106: 2170–2175. 19168634 10.1073/pnas.0806036106 PMC2630200

[19] Schaller MD (2001) Paxillin: a focal adhesion-associated adaptor protein. Oncogene 20: 6459–6472. 11607845 10.1038/sj.onc.1204786

[20] Turner CE, Deakin NO (2008) Paxillin comes of age. Journal of Cell Science 121: 2435–2444. 18650496 10.1242/jcs.018044 PMC2522309

[21] Digman MA, Brown CM, Horwitz AR, Mantulin WW, Gratton E (2008) Paxillin dynamics measured during adhesion assembly and disassembly by correlation spectroscopy. Biophysical Journal 94: 2819–2831. 17993500 10.1529/biophysj.107.104984 PMC2267137

[22] Lanzano L, Digman MA, Fwu P, Giral H, Levi M, et al (2011) Nanometer-scale imaging by the modulation tracking method. Journal of Biophotonics 4: 415–424. 21462350 10.1002/jbio.201100002 PMC3393040

[23] Levi V, Ruan QQ, Gratton E (2005) 3-D particle tracking in a two-photon microscope: Application to the study of molecular dynamics in cells. Biophysical Journal 88: 2919–2928. 15653748 10.1529/biophysj.104.044230 PMC1305386

[24] Kis-Petikova K, Gratton E (2004) Distance measurement by circular scanning of the excitation beam in the two-photon microscope. Microscopy Research and Technique 63: 34–49. 14677132 10.1002/jemt.10417

[25] Rossow M, Mantulin WW, Gratton E (2009) Spatiotemporal image correlation spectroscopy measurements of flow demonstrated in microfluidic channels. Journal of Biomedical Optics 14. 10.1117/1.3088203 PMC2702990 19405744

[26] Hebert B, Costantino S, Wiseman PW (2005) Spatiotemporal image correlation spectroscopy (STICS) theory, verification, and application to protein velocity mapping in living CHO cells. Biophysical Journal 88: 3601–3614. 15722439 10.1529/biophysj.104.054874 PMC1305507

[27] Tanner K, Ferris DR, Lanzano L, Mandefro B, Mantulin WW, et al (2009) Coherent Movement of Cell Layers during Wound Healing by Image Correlation Spectroscopy. Biophysical Journal 97: 2098–2106. 19804742 10.1016/j.bpj.2009.06.052 PMC2756390

[28] Digman MA, Brown CM, Sengupta P, Wiseman PW, Horwitz AR, et al (2005) Measuring fast dynamics in solutions and cells with a laser scanning microscope. Biophysical Journal 89: 1317–1327. 15908582 10.1529/biophysj.105.062836 PMC1366616

[29] Digman MA, Dalal R, Horwitz AF, Gratton E (2008) Mapping the number of molecules and brightness in the laser scanning microscope. Biophysical Journal 94: 2320–2332. 18096627 10.1529/biophysj.107.114645 PMC2257897

[30] Wolf K, Mazo I, Leung H, Engelke K, von Andrian UH, et al (2003) Compensation mechanism in tumor cell migration: mesenchymal-amoeboid transition after blocking of pericellular proteolysis. Journal of Cell Biology 160: 267–277. 12527751 10.1083/jcb.200209006 PMC2172637

[31] McGrath JL, Tardy Y, Dewey CF, Meister JJ, Hartwig JH (1998) Simultaneous measurements of actin filament turnover, filament fraction, and monomer diffusion in endothelial cells. Biophysical Journal 75: 2070–2078. 9746549 10.1016/S0006-3495(98)77649-0 PMC1299879

[32] Wolf K, Alexander S, Schacht V, Coussens LM, von Andrian UH, et al (2009) Collagen-based cell migration models in vitro and in vivo. Seminars in Cell & Developmental Biology 20: 931–941. 19682592 10.1016/j.semcdb.2009.08.005 PMC4021709

[33] Maskarinec SA, Franck C, Tirrell DA, Ravichandran G (2009) Quantifying cellular traction forces in three dimensions. Proc Natl Acad Sci U S A 106: 22108–22113. 20018765 10.1073/pnas.0904565106 PMC2799761

[34] Turner CE, Deakin NO (2011) Distinct roles for paxillin and Hic-5 in regulating breast cancer cell morphology, invasion, and metastasis. Molecular Biology of the Cell 22: 327–341. 21148292 10.1091/mbc.e10-09-0790 PMC3031464

[35] Rhee S (2009) Fibroblasts in three dimensional matrices: cell migration and matrix remodeling. Experimental and Molecular Medicine 41: 858–865. 19745603 10.3858/emm.2009.41.12.096 PMC2802681

[36] Pathak A, Kumar S (2011) Biophysical regulation of tumor cell invasion: moving beyond matrix stiffness. Integr Biol (Camb) 10.1039/c0ib00095g 21210057

[37] Grinnell F, Miron-Mendoza M, Seemann J (2010) The differential regulation of cell motile activity through matrix stiffness and porosity in three dimensional collagen matrices. Biomaterials 31: 6425–6435. 20537378 10.1016/j.biomaterials.2010.04.064 PMC2900504

[38] Friedl P, Wolf K (2010) Plasticity of cell migration: a multiscale tuning model. J Cell Biol 188: 11–19. 19951899 10.1083/jcb.200909003 PMC2812848

[39] Poincloux R, Collin O, Lizarraga F, Romao M, Debray M, et al (2011) Contractility of the cell rear drives invasion of breast tumor cells in 3D Matrigel. Proc Natl Acad Sci U S A 108: 1943–1948. 21245302 10.1073/pnas.1010396108 PMC3033302

