# Supplementary figures and images for: Nanoimaging of Focal Adhesion Dynamics in 3D
Source: PLoS One. 2014 Jun 24;9(6):e99896. doi: 10.1371/journal.pone.0099896 (PMC4069057; doi:10.1371/journal.pone.0099896)

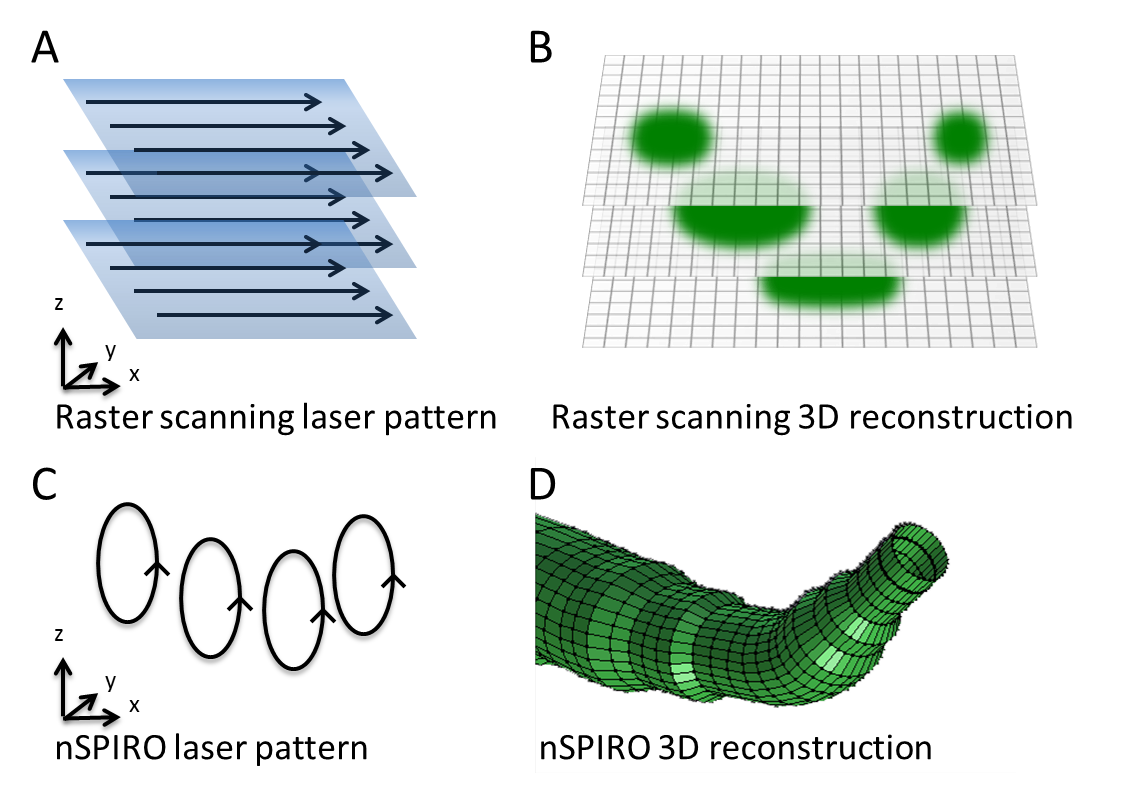

Supplement: Figure S1 — Comparison of raster scan and nSPIRO. (A) The raster scan pattern for 3D z-stack imaging. At each xy plane (blue), the laser beam scans line by line (black arrows) to acquire the intensity. Then the laser beam moves to a different z position and repeats the same raster scan pattern to acquire the image for another xy plane. (B) After the image is acquired as described from (A), the 3D image is reconstructed by stacking images of each xy plane at different z positions together. The grid represents pixels. As shown in this example, for a 3D sparse structure, there could be a large amount of pixels containing no information of the object of interest (green). (C) The orbital scanning pattern for nSPIRO 3D imaging. Instead of scanning line by line as raster scan, nSPIRO uses orbital laser pattern for image acquisition. With the feedback algorithm, the center of orbit scanning is adjusted to maintain the orbit on the object of interest. (D) nSPIRO orbital scan contains the information of structure size, which is used to create the mesh (black grids), and the intensity acquired along the orbit is painted on the mesh for image reconstruction. With this method, all the pixels acquired are from the object of interest, which increases the data acquisition efficiency compared to raster scan. (TIF) [file pone.0099896.s001.tif]
